# Editorial: Inflammation as a fundamental mechanism of stroke

**DOI:** 10.3389/fimmu.2026.1993470

**Published:** 2026-09-24

**Authors:** Alexander Brill, Simon F. De Meyer

**Affiliations:** 1 Department of Cardiovascular Sciences, College of Medicine and Health, School of Medical Sciences, University of Birmingham, Birmingham, United Kingdom; 2 Laboratory for Thrombosis Research, Department of Cardiovascular Sciences, KU Leuven Kulak, Kortrijk, Belgium

**Keywords:** stroke, inflammation, thromboinflammation, blood brain barrier, neutrophil extacellular traps

Stroke begins as a vascular event, but its biological consequences rapidly extend far beyond the site of vessel occlusion or rupture. Inflammatory responses develop within the thrombus, vascular wall and brain parenchyma, while simultaneously reshaping peripheral immunity and systemic physiology. These responses are neither uniformly detrimental nor simply secondary to tissue injury. Depending on their timing, anatomical compartment and cellular context, immune pathways can promote thrombosis, blood-brain barrier (BBB) disruption and neuronal death, but can also contribute to debris clearance, vascular repair and functional recovery. The Research Topic “Inflammation as a fundamental mechanism of stroke” was conceived to capture this complexity and to bring together work spanning thromboinflammation, innate and adaptive immunity, neurovascular injury, systemic immune reprogramming, inflammatory resolution, biomarkers and emerging therapeutic approaches.

Several contributions emphasize that inflammation in stroke begins at, or even before, the neurovascular interface. Littleton et al. examined human thrombi retrieved by mechanical thrombectomy and identified neutrophil α-defensin-1 together with abundant neutrophil extracellular traps (NETs); their *in vitro* observations further support a potential link between neutrophil degranulation and NETosis. Ardic and Dinc extend this concept in a review of reciprocal interactions between NETs and microglia/macrophages, describing a feed-forward circuit in which neutrophil products activate resident myeloid cells, while inflammatory signalling promotes further neutrophil recruitment and NET formation. Importantly, thromboinflammation is also relevant before an acute cerebrovascular event. Bai et al. identify aminopeptidase N (ANPEP) as a regulator of lipid handling and foam-cell formation in macrophages associated with large-artery atherosclerotic stroke. Together, these studies place inflammatory cells not merely downstream of cerebral ischemia, but within the vascular and thrombotic processes that determine whether, where and how stroke occurs.

Once cerebral injury is established, the BBB becomes both a target and an active participant in inflammation. Wang et al. review the reciprocal relationship between immune activation and BBB disruption after ischemic stroke, highlighting how microglia, infiltrating leukocytes, inflammatory mediators and endothelial dysfunction can create a self-amplifying cycle of barrier failure and tissue injury, while also acknowledging the reparative functions of immune responses at later stages. Wu et al. provide a complementary mechanistic perspective in intracerebral hemorrhage, identifying annexin A2 (ANXA2) as an upstream contributor to NLRP3 inflammasome activation, gasdermin-D-associated pyroptosis and inflammatory injury. At a systems level, Xu et al. integrate transcriptomic datasets and machine learning approaches across experimental cerebral ischemia models to identify conserved immune-associated transcriptional regulators, with Cebpa and Relb linked particularly to microglial populations. These contributions collectively illustrate that vascular permeability, innate immune sensing, regulated cell death and transcriptional reprogramming are interconnected rather than independent components of post-stroke inflammation.

A second major theme is that the immune response to stroke is not confined to the brain. Kim et al. demonstrate enhanced splenic myelopoiesis after experimental stroke and show that inhibition of the inflammatory alarmins S100A8/A9 attenuates this response and improves outcome, highlighting the spleen as an active source and regulator of post-stroke myeloid responses. At the adaptive immune level, Gellrich et al. explore manipulation of T-cell metabolism and polarization using the acetyl-CoA carboxylase inhibitor soraphen A. Their findings illustrate the possibility of shifting the balance between inflammatory T-helper responses and regulatory T cells, while also emphasizing that such interventions must be considered in the context of infection. This point is central to the review by Ascaso-Vidal et al., who revisit post-stroke immunosuppression and argue that it cannot be explained solely by classical sympathetic and hypothalamic-pituitary-adrenal pathways. Instead, damage-associated signals, altered hematopoiesis, neutrophil activation and lymphocyte dysfunction contribute to a complex systemic immune phenotype.

This systemic perspective is developed further by Gao et al., who frame stroke as a disruption of neuroimmune regulatory circuitry connecting neural afferent pathways, central autonomic and endocrine control, and peripheral immune organs. Such a framework helps reconcile two apparently opposing features of stroke biology: intense local inflammation and simultaneous systemic immunosuppression. Zhou et al. similarly consider stroke through an integrated neuro-endocrine-immune lens in their review of acupuncture, discussing how neural, endocrine and immune pathways may be modulated in parallel. Irrespective of the intervention considered, these contributions make an important conceptual point: post-stroke immunity is distributed across organs and regulatory networks, and approaches focused exclusively on inflammatory events within the infarcted brain are unlikely to capture the full biology of the disease.

The translational challenge is therefore not simply to establish whether inflammation is “high” or “low”, but to define which inflammatory state is present, in which patient, and at what time. Two clinical studies in this Research Topic address this need using readily accessible systemic biomarkers. Zhu et al. report that a higher low-grade inflammation score is associated with worse neurological outcomes in patients with acute pontine infarction. Şahin and Şahin compare composite inflammatory indices across ischemic and hemorrhagic stroke and identify the C-reactive protein-albumin-lymphocyte index as a potentially informative prognostic measure, while emphasizing the need for further validation. These studies complement molecular approaches such as the cross-species transcriptional analysis of Xu et al. and point toward a future in which inflammatory phenotyping may help stratify patients rather than treating stroke-associated inflammation as a single biological entity.

The inclusion of both ischemic and hemorrhagic stroke also highlights an important balance between shared mechanisms and disease-specific context. Leukocyte activation, inflammasome signalling, BBB dysfunction and systemic inflammatory responses occur across stroke subtypes, but their triggers and temporal relationships differ substantially. In ischemic stroke, inflammation develops in the setting of arterial occlusion, hypoxia-reperfusion and frequently an inflammatory thrombus or atherosclerotic substrate; after intracerebral hemorrhage, blood components, hematoma products and mechanical tissue injury provide a different initiating environment. The mechanistic findings of Wu et al. in hemorrhage and the cross-subtype clinical analysis of Şahin and Şahin therefore caution against assuming that an inflammatory biomarker or therapeutic target will behave identically across all forms of stroke. Shared pathways may provide common therapeutic opportunities, but they will require validation within the specific vascular and clinical setting in which they are to be used.

The therapeutic studies and reviews within the collection also reflect a shift away from indiscriminate suppression of inflammation. Wang et al. discuss specialized pro-resolving mediators, including lipoxins, resolvins, protectins and maresins, as active regulators of the transition from inflammation toward repair. This distinction between anti-inflammation and pro-resolution is particularly important in stroke, where complete suppression of immune activity may interfere with host defence, clearance of damaged tissue and later recovery. Other contributions explore distinct routes to modulating inflammatory injury. Lu et al. describe calcium sulfide nanoparticles as sustained hydrogen-sulfide donors with neuroprotective and anti-inflammatory effects in experimental ischemic stroke. Wu et al. combine network pharmacology with experimental validation to investigate astragaloside IV, reporting effects on inflammatory signalling, BBB integrity and cerebral ischemia-reperfusion injury. Together with the immunometabolic strategy investigated by Gellrich et al., these studies illustrate the diversity of potential entry points into the inflammatory cascade, from intracellular signalling and cellular metabolism to resolution pathways and drug-delivery technologies.

Taken together, the 16 articles support several broader conclusions. First, inflammation in stroke is spatially distributed: it operates within atherosclerotic lesions and thrombi, at the cerebral endothelium and BBB, in the injured brain, and in peripheral immune organs. Second, it is temporally dynamic. The same cell population or mediator may have different consequences during vascular occlusion, acute tissue injury, systemic immune adaptation and later repair. Third, apparently distinct processes such as thrombosis, inflammasome activation, NET formation, BBB failure, myelopoiesis and lymphocyte dysfunction, are mechanistically coupled and can reinforce one another. Finally, the coexistence of damaging inflammation, compensatory immunosuppression and reparative immune functions makes a simple “pro-inflammatory versus anti-inflammatory” classification increasingly inadequate.

These observations have direct implications for translation. Successful immunomodulation in stroke will likely require attention to disease subtype, timing, anatomical compartment, concomitant infection and the baseline immune state of the patient. Biomarkers that identify relevant inflammatory endotypes will be needed to accompany therapies, and experimental studies should increasingly integrate vascular, neural and systemic readouts rather than focusing on a single compartment. Human thrombectomy material, longitudinal blood profiling, advanced imaging, transcriptomics and other multi-omic approaches provide complementary opportunities to connect mechanistic experimental work with patient biology. Equally important will be distinguishing interventions that suppress damaging inflammatory amplification from those that actively promote resolution and repair.

The contributions to this Research Topic therefore reinforce the view that inflammation is not an accessory feature of stroke but a fundamental mechanism linking vascular pathology, thrombus biology, neurovascular injury, neuronal damage and systemic responses. At the same time, they underscore why broadly anti-inflammatory strategies have been difficult to translate: “inflammation” encompasses multiple processes with different locations, kinetics and functions. A more productive direction is precision immunomodulation: targeting specific inflammatory mechanisms at defined stages while preserving protective immunity and enabling resolution. By integrating vascular, cerebral and systemic perspectives, the studies collected here help move the field toward that more nuanced framework and identify both mechanistic questions and translational opportunities for the next generation of stroke research.

